# Bilateral Nasolabial Cysts Mimicking Inferior Turbinate Hypertrophy in a Patient With Sinonasal Polyposis

**DOI:** 10.7759/cureus.103058

**Published:** 2026-02-05

**Authors:** Salah M Mahmoud, Ahmed Shaikh, Hamad Al Saey

**Affiliations:** 1 Otolaryngology - Head and Neck Surgery, Sidra Medicine, Doha, QAT; 2 Otolaryngology - Head and Neck Surgery, Hamad Medical Corporation, Doha, QAT

**Keywords:** bilateral, case report, endoscopic excision, inferior turbinate hypertrophy, nasolabial cyst, sinonasal polyposis

## Abstract

Nasolabial cysts are rare, nonodontogenic cysts of the anterior nasal floor and nasolabial fold. Bilateral presentation is uncommon and may mimic inferior turbinate hypertrophy, leading to misdiagnosis. A 41-year-old Qatari man presented with a five-year history of persistent nasal obstruction, headache, and nasal discharge. He had chronic sinonasal polyposis and a prolonged history of topical nasal decongestant use, with poor response to intranasal steroids. Two CT scans performed three years apart were initially reported as showing sinonasal polyposis; however, retrospective review revealed subtle, bilateral, well-circumscribed anterior cystic lesions inferior to the inferior turbinates that had been overlooked. During functional endoscopic sinus surgery, symmetric bilateral nasolabial cysts measuring 1.5-2.5 cm were identified and excised endoscopically. Histopathology confirmed respiratory epithelium-lined cysts. Postoperative recovery was favorable, with no recurrence at 4.5 months. Bilateral nasolabial cysts are rare and can clinically and radiologically mimic inferior turbinate hypertrophy, especially in patients with rhinitis or sinonasal polyposis. Careful review of the anterior nasal cavity on imaging can prevent misdiagnosis. Endoscopic excision is both diagnostic and curative.

## Introduction

Nasolabial cysts, also called nasoalveolar or Klestadt’s cysts, are uncommon, nonodontogenic lesions of the anterior nasal floor and can resemble more common causes of nasal obstruction, including inferior turbinate hypertrophy, which may lead to misdiagnosis [1]. Although generally unilateral, bilateral cysts have been reported but remain rare, with only a limited number of cases described in the literature [2,3]. Because of their anterior and inferolateral location, they may be overlooked clinically, especially in patients with coexisting sinonasal pathology. We present a case of bilateral nasolabial cysts discovered intraoperatively after being overlooked on two CT scans in a patient with chronic sinonasal polyposis.

## Case presentation

Patient information

A 41-year-old Qatari male followed at Hamad Medical Corporation, Doha, presented with a five-year history of persistent nasal obstruction, stiffness, headache, and nasal discharge. He was a smoker and had a history of chronic rhinitis and sinonasal polyposis. He reported poor compliance with intranasal corticosteroid sprays and long-term use of topical nasal decongestants (xylometazoline), with minimal symptomatic relief.

Clinical findings

Examination revealed a deviated nasal septum to the left with a right bony spur, moderate-to-severe inferior turbinate hypertrophy, congested mucosa, and right-sided grade 2 nasal polyps. The vestibular aspect appeared consistent with normal inferior turbinates. Laboratory evaluation showed negative IgE and Phadiatop results.

Imaging

CT imaging of the paranasal sinuses performed in June 2021 demonstrated diffuse mucosal thickening with obliteration of the left frontal sinus and posterior ethmoid air cells, involvement of the ostiomeatal complexes and sphenoethmoidal recesses, hyperdense mucosal areas suggestive of allergic fungal sinusitis, and a right sphenoid sinus air-fluid level. Septal deviation with a right bony spur was also noted, and the impression was sinonasal polyposis.

A CT scan of the head performed in June 2024 for headache evaluation showed chronic pansinusitis with hyperdense areas suggestive of fungal involvement, along with persistent septal deviation and bony spur. No intracranial abnormality was identified.

**Figure 1:**
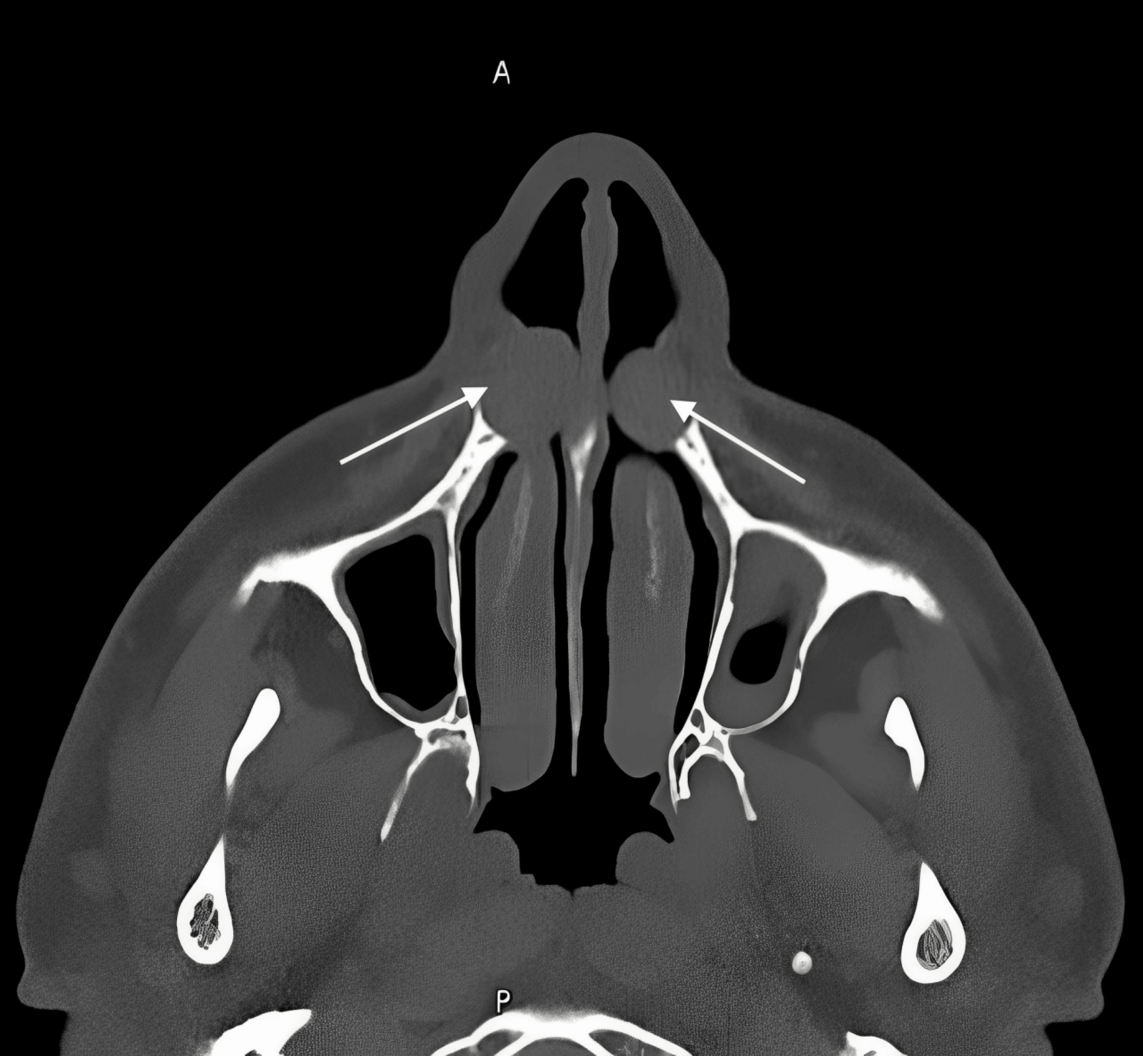
Axial CT of the anterior nasal cavity (performed on June 6, 2024) showing bilateral, well-circumscribed, low-attenuation submucosal lesions along the anterior nasal floor

**Figure 2:**
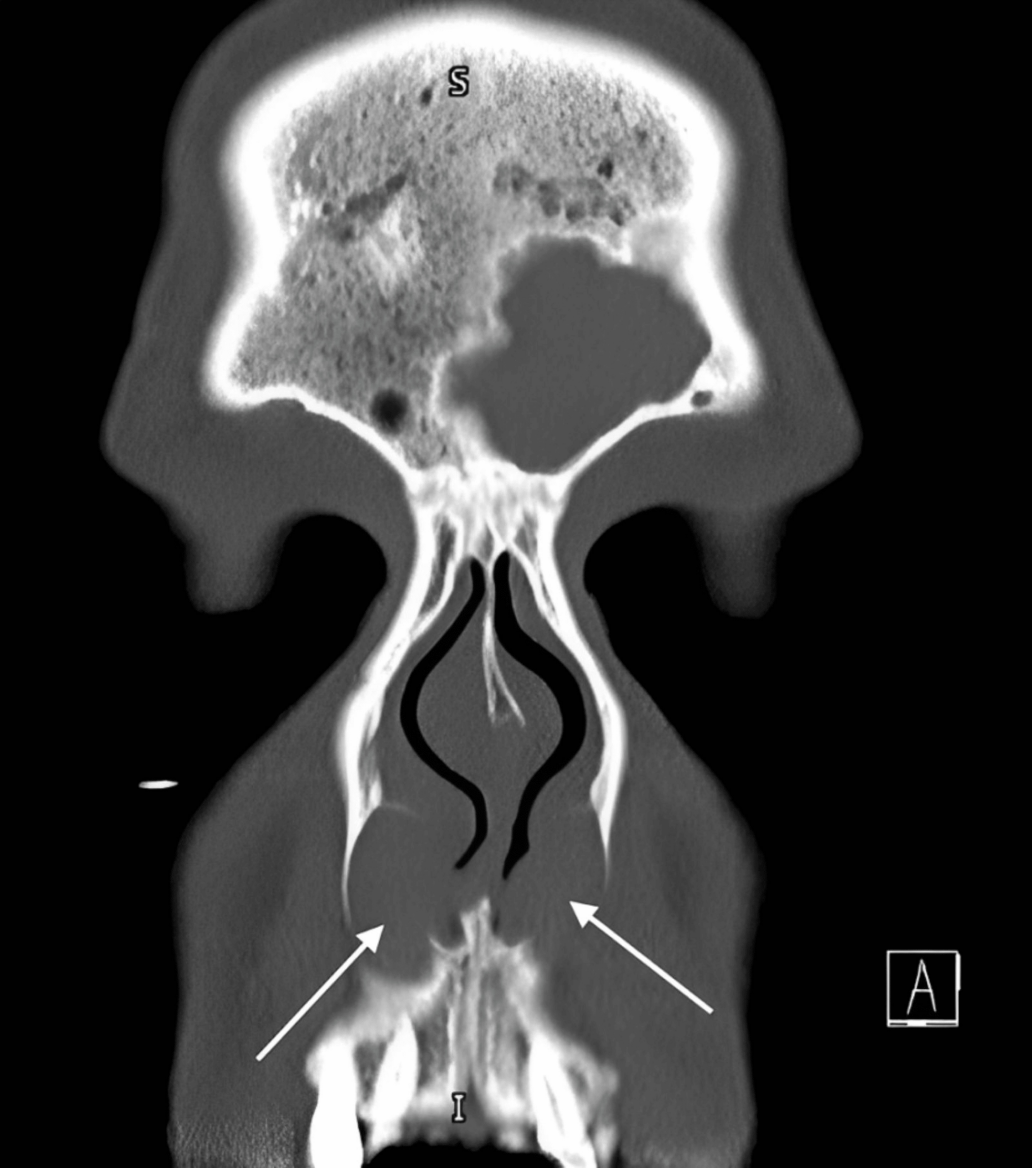
Coronal CT (soft-tissue window) of the anterior nasal cavity (performed on June 6, 2024) showing bilateral, well-circumscribed, low-attenuation submucosal lesions along the anterior nasal floor

On retrospective review, both CT examinations demonstrated subtle, symmetrical, well-circumscribed, low-attenuation submucosal lesions along the anterior nasal floor, lateral to the inferior turbinates and beneath the nasal alar region (Figure 1, Figure 2). These lesions were associated with mild scalloping and remodeling of the adjacent posterior maxillary bone, consistent with nasolabial cysts.

Operative findings

**Figure 3:**
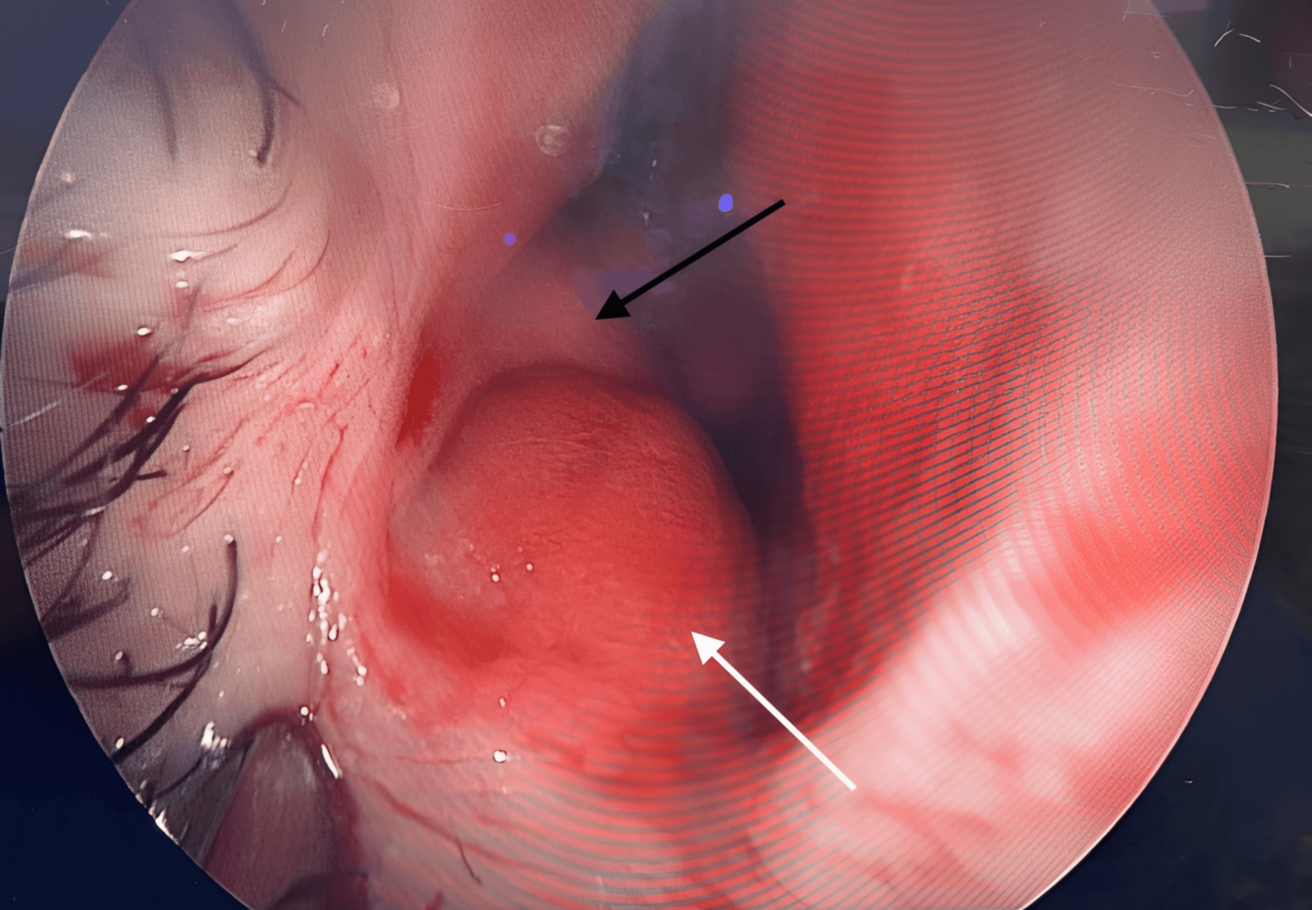
Intraoperative photograph during direct right nasal endoscopy (captured on April 15, 2025) demonstrating a smooth, glistening submucosal bulge at the anterior nasal floor (white arrow), just medial to the heads of the inferior turbinates (black arrow)

**Figure 4:**
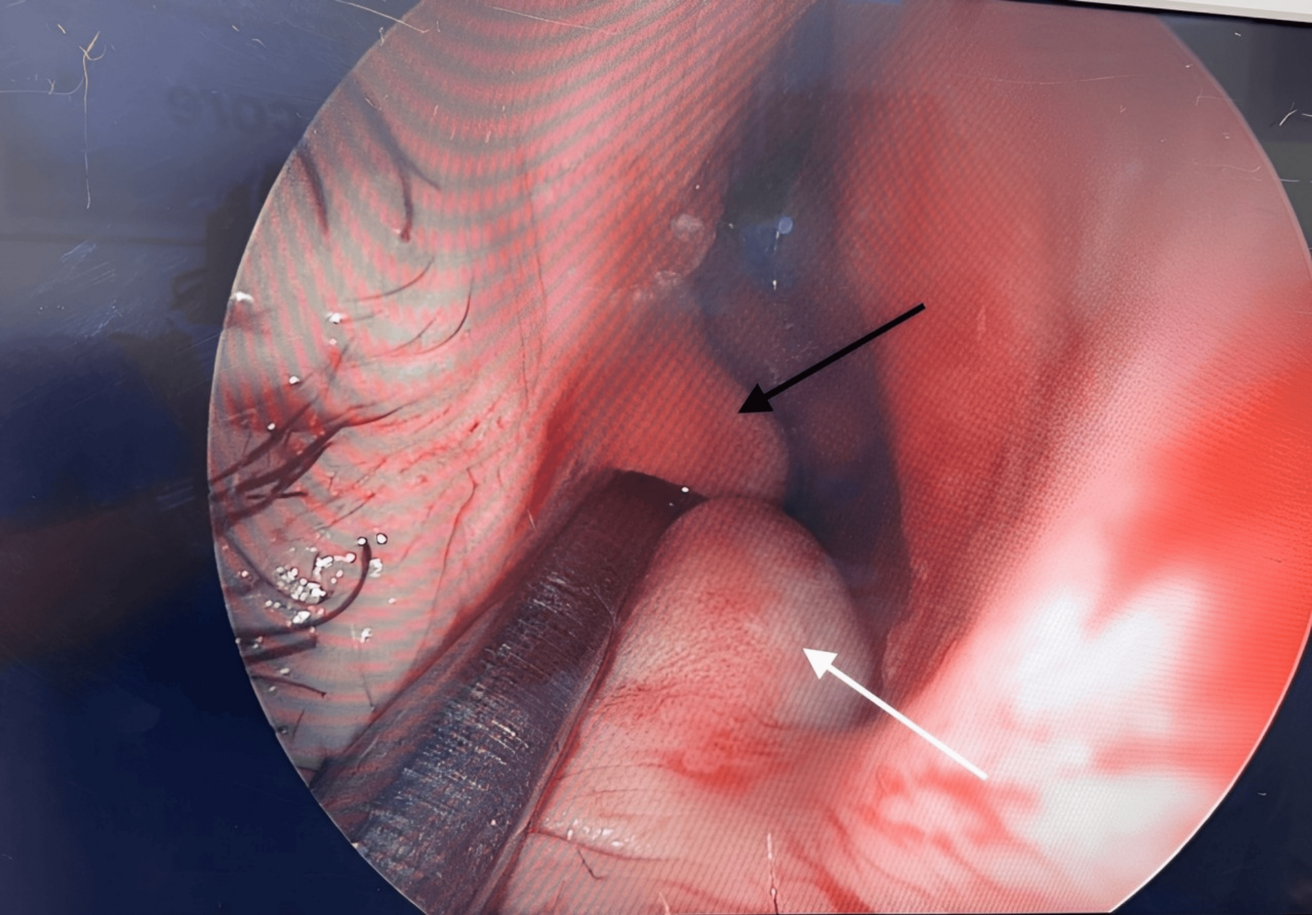
Intraoperative photograph during direct right nasal endoscopy (captured on April 15, 2025) showing the separation between the cyst (white arrow) and the right inferior turbinate (black arrow)

**Figure 5:**
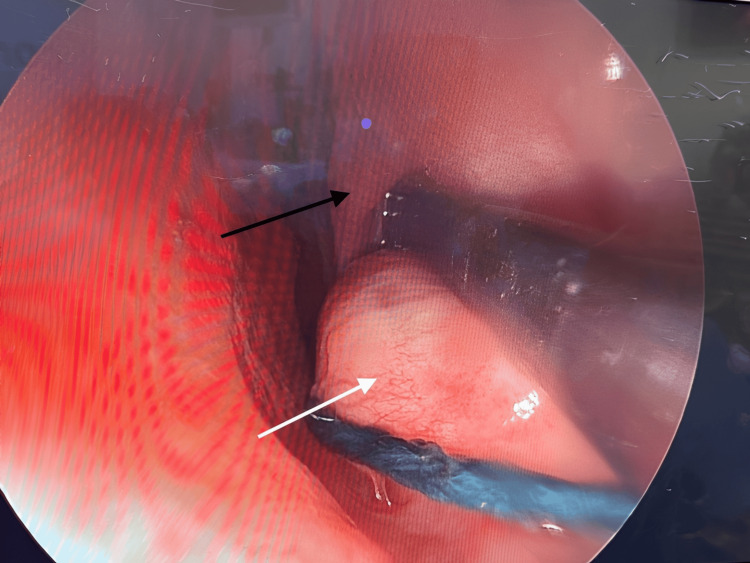
Intraoperative photograph during direct left nasal endoscopy (captured on April 15, 2025) showing the separation between the cyst (white arrow) and the left inferior turbinate (black arrow)

Bilateral transnasal endoscopic marsupialization and excision of the cysts were performed concurrently with functional endoscopic sinus surgery and septoplasty. The cysts were clearly distinct from the inferior turbinates, septum, and surrounding structures (Figure 3, Figure 4, Figure 5). Each cyst measured approximately 1.5-2.5 cm, projecting medially into the inferior nasal cavity. The swellings were tense, smooth, and glistening, with intact mucosa and no evidence of infection. They were clearly separate from the inferior turbinates, septum, paranasal sinuses, and nasopharynx.

Pathology

**Figure 6:**
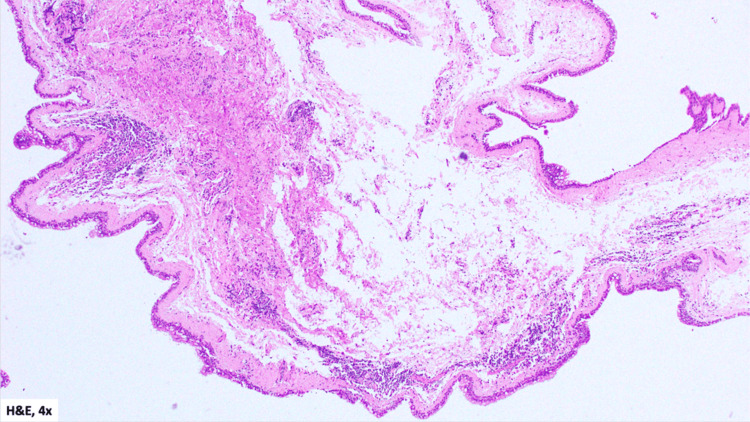
H&E-stained histopathology section from the patient’s specimen (obtained on April 15, 2025), viewed at low-power (4×) magnification, demonstrating overall tissue architecture

**Figure 7:**
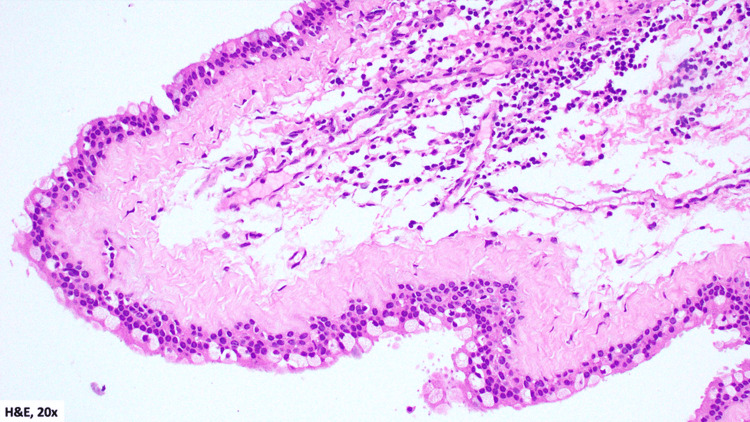
H&E-stained histopathology section from the patient’s specimen (obtained on April 15, 2025), viewed at 20× magnification, showing a cyst wall lined by pseudostratified ciliated columnar (respiratory-type) epithelium with goblet cells overlying a fibrous stroma, without chronic inflammation

Gross examination revealed multiple soft tissue fragments measuring 2.5 × 1.3 cm in aggregate. Microscopic examination demonstrated cyst walls lined by pseudostratified ciliated columnar (respiratory-type) epithelium with goblet cells, overlying a fibrous stroma with no significant inflammation (Figure 6, Figure 7). These findings were consistent with nasolabial cysts.

Follow-up and outcomes

The patient experienced nasal crusting in the early postoperative period but had no septal perforation. At five weeks postoperatively, he presented with epistaxis due to bleeding from the posterior septal branch of the right sphenopalatine artery, which was successfully managed under general anesthesia. At 4.5-month follow-up, there was no recurrence, and symptoms had improved substantially.

## Discussion

Nasolabial cysts typically present in the fourth or fifth decades, more commonly in women, and usually measure 1.5-3 cm. Early surgical reports describe their varied presentation and management options [4], while broader reviews have outlined their clinical features and diagnostic challenges [5]. Radiologically, typical CT findings include well-circumscribed, low-attenuation lesions at the nasal floor or lateral vestibule [6], and reference imaging sources highlight the importance of assessing the anteroinferior nasal cavity to avoid overlooking these lesions [7].

A systematic review of 311 patients found that bilateral cysts accounted for approximately 10.9% of reported cases, confirming their rarity and variability across presentations [8,9]. Narrative reviews have emphasized that misdiagnosis as hypertrophic turbinates or allergic rhinitis is a recurrent issue in clinical practice [10].

Histologically, nasolabial cysts are lined by respiratory epithelium, often containing goblet cells, consistent with the findings in our patient [11]. Historically, sublabial excision has been the standard approach [11,12], but more recent evidence supports endoscopic transnasal marsupialization as a minimally invasive alternative with comparable success and lower morbidity [4]. In this case, endoscopic excision resulted in complete removal with histologic confirmation, and no recurrence was observed at 4.5 months.

In this patient, prolonged medical therapy with topical corticosteroids and decongestants resulted in minimal improvement. This can be explained by the presence of a fixed mechanical obstruction caused by bilateral nasolabial cysts, rather than isolated mucosal hypertrophy. The coexistence of sinonasal polyposis likely contributed to diagnostic anchoring, leading to attribution of symptoms solely to turbinate hypertrophy and delayed recognition of the cysts.

Although rare, nasolabial cysts should be considered in the differential diagnosis of apparent inferior turbinate hypertrophy, particularly when the enlargement is focal, rounded, and located anteriorly. Careful review of the anterior nasal cavity on CT is essential, and both radiologists and surgeons should maintain a high index of suspicion to avoid misdiagnosis. Bilateral involvement, while uncommon, is well documented in the literature and warrants systematic inspection of both sides. Endoscopic excision provides an effective and safe treatment option while also allowing definitive histopathological diagnosis.

## Conclusions

Bilateral nasolabial cysts are rare lesions that can mimic inferior turbinate hypertrophy and contribute to persistent nasal obstruction. In patients with sinonasal polyposis, these cysts may be masked and overlooked on routine imaging. Thorough endoscopic and radiologic assessment of the anterior nasal floor is essential for accurate diagnosis. Endoscopic excision is safe, minimally invasive, and provides definitive treatment with excellent outcomes.
